# ClonorESTdb: a comprehensive database for *Clonorchis sinensis* EST sequences

**DOI:** 10.1186/1756-0500-7-388

**Published:** 2014-06-24

**Authors:** Dae-Won Kim, Won Gi Yoo, Sanghyun Lee, Myoung-Ro Lee, Yu-Jung Kim, Shin-Hyeong Cho, Won-Ja Lee, Jung-Won Ju

**Affiliations:** 1 Division of Malaria and Parasitic Diseases, Centre for Immunology and Pathology, Korea National Institute of Health, Chungbuk 363-951, Republic of Korea

**Keywords:** *Clonorchis sinensis*, Expressed sequence tags (ESTs), Transcriptome, Database

## Abstract

**Background:**

Clonorchiasis, which is primarily caused by liver fluke (Platyhelminthes), is a fatal infectious disease that is mainly associated with bile duct malignancy and the subsequent development of cholangiocarcinoma. Thus, a genomic approach now represents an important step to further our knowledge of biology and the pathology of these parasites. The results of expressed sequence tags (ESTs) sequencing need to be well organized into databases to provide an integrated set of tools and functional information.

**Findings:**

Here, the ClonorESTdb database represents a collection of *Clonorchis sinensis* ESTs that is intended as a resource for parasite functional genomics. A total of 55,736 successful EST sequences, which are cleaned and clustered into non-redundant 13,305 *C. sinensis* assembled EST sequences (6,497 clusters and 6,808 singletons), were obtained from three in-house prepared cDNA libraries of *C. sinensis* at different developmental stages. The assembled consensus sequences were annotated using the BLAST algorithm or/and hmm against NCBI NR, UniProt, KEGG and InterProScan. The ClonorESTdb database provides functional annotation, their expression profiles, tandem repeats and putative single nucleotide polymorphisms with utility tools such as local BLAST search and text retrieval.

**Conclusions:**

This resource enables the researcher to identify and compare expression signatures under different biological stages and promotes ongoing parasite drug and vaccine development and biological research.

**Database URL:**http://pathod.cdc.go.kr/clonorestdb/

## Findings

*Clonorchis sinensis* is the human liver fluke of the class Trematoda (phylum Platyhelminthes: Digenea). The human host is infected by consuming raw and inadequately cooked freshwater fish with *C. sinensis* metacercariae. Clonorchiasis is a common infectious disease in many eastern Asian countries, including Korea, China, Japan and Vietnam. It is estimated that Clonorchiasis affects approximately 35 million people worldwide [1] and more than 600 million people are at risk of infection in East Asia and Eastern Europe [2].

Metacercariae of *C. sinensis* exist in the small intestine and the juvenile worms migrate up through the Ampulla of Vater and the common bile duct [3]. The livers of patients with clonorchiasis appear almost normal in cases of light infections, but slightly dilated and thickened peripheral bile ducts are present in cases of heavy infections. Patients with clonorchiasis persistently suffer from fatigue, jaundice, abdominal distress and indigestion [4]. Chronic infection can cause several hepatobiliary disease manifestations, such as cholangitis, cholecystitis, cholelithiasis, hepatomegaly and fibrosis of the periportal tract [5-7]. Although *C. sinensis* is officially recognized as a biological human group I carcinogen by the International Agency for Research on Cancer (IARC) and the World Health Organization (WHO) [8], the molecular and cellular biology of *C. sinensis* has been significantly underexplored due to the lack of genomic database resources from well-isolated full-length cDNAs.

The advent of next-generation sequencing machines that involve GS 454 [9], Solexa [10] and SOLiD [11] are revolutionizing molecular biology by generating hundreds of thousands of sequencing reads in parallel. The genome and transcriptome sequences of a growing number of model organisms have been published in recent years, which have drawn new insights into parasite research [12,13]. However, the *de novo* large-scale sequencing of a non-model parasite is still a laborious task and an interesting challenge. Expressed sequence tags (ESTs) are a cost effective alternative and a powerful tool that provides sufficient information about functional proteins. In particular, the ESTs from a full-length cDNA library allow researchers to be cloned and provide material sources so that many intriguing biological issues can be isolated.

The aim of this study is to provide important database resources for the characterization and understanding of the functional genes of *C. sinensis*. Here, we have constructed and described ClonorESTdb, a web-based ESTs database resource that involves systematic functional annotation, which comprises more than 55,736 high-quality ESTs based on three full-length enriched cDNA libraries. The ESTs obtained were assembled into 13,305 *C. sinensis* Assembled EST sequences (CsAEs) comprising 6,497 clusters and 6,808 singletons by aligning CsAEs onto the non-redundant public NCBI NR database, UniProt, KEGG, InterProScan and Gene Ontology (GO). The ClonorESTdb database described here provides key insights into the differential gene expression of *C. sinensis* in a range of developmentally relevant conditions.

### Database architecture

The ClonorESTdb database runs on a RedHat Enterprise Linux 5.5 platform with the Apache web server version 2.2. We also used the relational Oracle database 11 g standard version to develop and support an integrated database schema for storing sequence data, preprocessed data and final functional annotation. The web application was implemented with JSP (Java Server Pages), JavaServelet technology and the AJAX framework. The web interfaces were designed using HTML language with some scripts in JavaScript and the pages utilized cascading style sheet (CSS) properties. The database is currently optimized to work best with Microsoft Internet Explorer 8 (optimal resolution 1024 × 800).

### Data source

We constructed full-length cDNA libraries (adult, metacercaria and egg) from *C. sinensis* and generated large-scale 60,768 ESTs data by 5’-end sequencing of individual clones [14]. All of the raw and cleaned data can be downloaded from the ClonorESTdb database.

### The pipeline for constructing the database

In our study, a total of 55,736 *C. sinensis* EST sequences that were derived from three cDNA libraries (adult, metacercaria and egg) were used to construct the database. To analyze the data, we developed a pipeline for the ClonorEST Project divided into three steps: sequence cleaning, sequence clustering and assembly, and automatic annotation.

#### ***Sequence cleaning***

**Figure 1:**
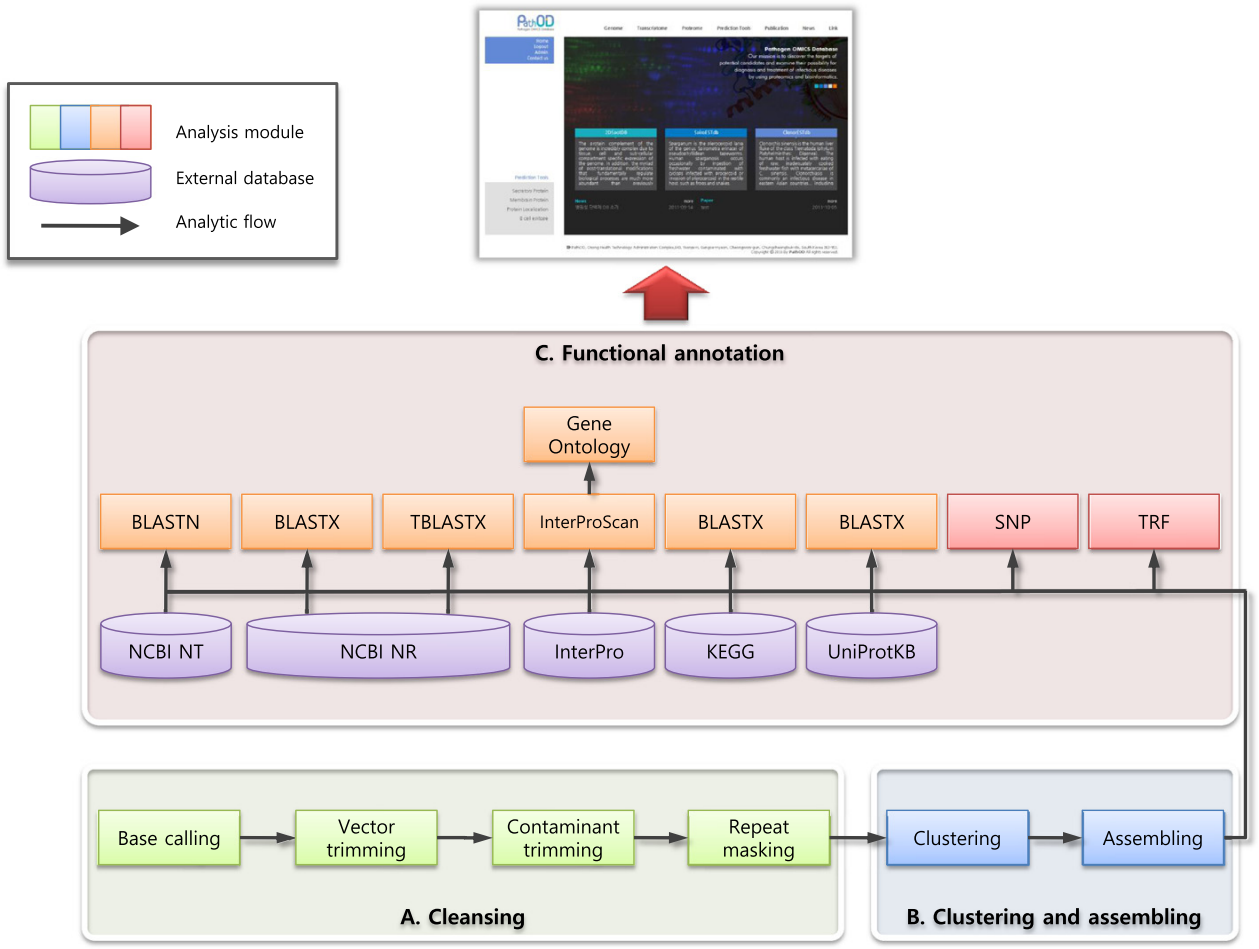
**Workflow scheme of an EST analytic process. A**. The cleaning step is composed of base calling (Phred), vector masking (cross_match), contaminant trimming (SeqClean) and repeat masking (RepeatMasker). **B**. The cleaned EST sequences are sent to the next step: clustering and assembling (TGICL). **C**. CsAEs sequences are aligned with various public databases for assigning their putative function and predicting structural variation (AutoSNP and TRF).

Cleaning is an important part of processing and is used to obtain high-quality EST datasets from raw EST sequences. After base calling was performed using Phred [15], the cleaning process implemented Cross_match (version 0.990329) for masking any vector and contaminant sequences, SeqClean (http://seqclean.sourceforge.net/) for eliminating undetermined bases, poly(A) tails and low complexity elements, RepeatMasker (http://www.repeatmasker.org/) for removing interspersed repeats, such as SINEs (short interspersed nuclear element), LINEs (long interspersed nuclear elements), LTRs (long terminal repeat) and DNA elements included in the Repbase repetitive element library (http://www.girinst.org/) [16] (Figure 1A).

#### ***Sequence clustering and assembly***

The clustering procedure was a basic step that was used to collect overlapping CsAEs sequences that originated from the same transcript of a single gene; this is performed to reduce redundancy. The assembly procedure is executed to align and merge many overlapping EST sequences of a much longer DNA sequence to reconstruct a putative full-length transcript sequence. For clustering and assembling, we used TGICL to create a grouping EST sequences and CAP3 for assembling the clustered EST sequences [17,18] (Figure 1B).

#### ***Automatic functional annotation***

For more accuracy and further variety of functional annotation, we used various annotation algorithms and public databases. First, we assigned putative functions to the CsAEs based on BLASTN (Query Coverage ≥ 80.0%, Identity ≥ 70.0%, E-value ≤ 1.0e-5), BLASTX and TBLASTX (Match No. ≥ 30 aa, Identity ≥ 25.0%, E-value ≤ 1.0e-5) searches against the GenBank NT and NR databases (ftp://ftp.ncbi.nih.gov/blast/db/FASTA/). Second, metabolic pathways are extremely important for correctly inferring pathogen invasion, host defense, adaptation, pathogen life cycle and host-pathogen interactions. To get a rationale for the development of anti-parasitic drugs and vaccines, we must identify all parasite-specific metabolic pathways. To identify the pathways, additional annotations were created against the UniProtKB database (http://www.ebi.ac.uk/uniprot) and the KEGG database (http://www.genome.jp) using BLASTX (Match No. ≥ 30 aa, Identity ≥ 25.0%, E-value ≤ 1.0e-5). All BLAST algorithms were implemented using a TimeLogic DeCypher system (Active Motif, Inc., http://www.activemotif.com). We also used the InterProScan tool to extract additional functional domains (E-value ≤ 1.0e-4). To gain a better classification of the biological function of the CsAEs, an analysis of the functions was conducted using GO terms according to three categories: molecular function, biological process and cellular component. We used Tandem Repeats Finder (TRF) and AutoSNP to detect structural variations [19,20] (Figure 1C).

## Utility and discussion

The aims of this database project included 1) the construction of an integrative database of the *C. sinensis* transcriptome, 2) the maintenance of a well-organized functional annotation and the identification and characterization of factors such as parasite specific antigen and 3) data-mining for tissue-specific expression to discover key pathways related to parasite proliferation that are essential to its maintenance. The database presented here and named ClonorESTdb consists of 55,736 EST entries from *C. sinensis* at three stages including adult, metacercaria and egg.

### ClonorESTdb web interface

The ClonorESTdb provides a user-friendly interface with the seven following options in the main menu: (1) a detailed pre-processing report, (2) clustering and assembling report and viewer, (3) various functional annotation report and (4) download all of the raw data and the analyzed result. The data can be viewed at various degrees of detail, either as an overview (a list of search results) or as a detailed results page for a selected sequence and functional annotation.

### Pre-processing and assembling report

**Figure 2:**
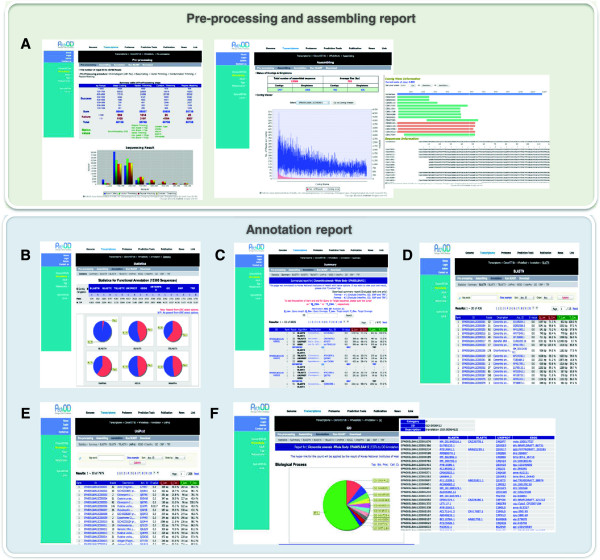
**Snapshots of the ClonorESTdb web application.** The panels show examples for various general functions. **A**. An example of a pre-processing report, which shows the cleaning sequences, assembly status, contig view and trace view. **B**. A statistics page showing an annotation result. **C**. A summary page showing various annotation results. **D**. A BLASTX annotation report page. **E**. A UniProt annotation report page. **F**. A GO annotation report page.

After pre-processing, all of results are stored in a database for evaluation and reporting, according to four steps (base calling, vector trimming, contamination, trimming and repeat masking). The pre-processing shows a summary table of the pre-processing steps. The assembly report of ClonorESTdb provides a comprehensive summary of the status of contigs and singletons, such as sequencing status and clustering information. Furthermore, each sequence and each contig were assigned a detailed on-the-fly page where the ID of the nucleotide sequence can be clicked. In each contig page, the graphical display of the contigs and the contig alignment sequence information are provided. We also provide the module of a trace viewer that allows users to evaluate the result of the sequencing of raw data that was derived from the machine on the web (Figure 2A).

### Annotation report

For various functional annotation reporting, the online database of ClonorESTdb provides a comprehensive information system for analyzing data from the 55,736 EST sequencing project. An annotation report provides 12 categories of analysis information to allow users to easily monitor all of the analyzed EST datasets. The “statistics” page provides a numeric table of functional annotation and the annotated species distribution derived from all the algorithms (Figure 2B). On the “summary report” page, users can see all of the detailed annotation information regarding how the CsAEs were annotated in the system and the users can download entire annotation files as Excel files (Figure 2C). The annotation results against various databases contain clone and contig information, including the sequence, putative identification, annotation detailed information, a link to the nearest homologue in the public database, the length of the homologous sequence and the percentage identity of the nearest match. In addition, existing information for all CsAEs in an EST set can be retrieved using full-text matching (against read id, consensus id, gene name, gene accession number, functional description and E-value score) from the search tool (Figure 2D). In this section, ClonorESTdb provides access to the enzyme and pathway information in UniProt and KEGG linked to an external database. Users can mine the enzyme information from the annotated results of KEGG and UniProt databases that contained an EC number in the description (Figure 2E). Here, for the task of representing and processing information by domain analysis in the “InterPro” page, their products and their functions are presented in GO categories (Figure 2F). When working on one or more given individuals or species, the biologist may wish to search for markers in intra-specific polymorphisms or intra-specific polymorphisms. Thus, we also added single nucleotide polymorphisms (SNPs) and simple tandem repeats (STRs) data.

## Conclusion

In summary, ClonorESTdb is the first incorporated online transcriptome database of *C. sinensis* that can be freely accessed and downloaded. Moreover, this database provides various utility functions for similarity searches, transcriptome statistic information and investigation of user-supplied genomics and transcriptome sequences. The increasing amounts of genomics data that are derived from the advent of high-throughput technology will further stimulate the integration of ClonorESTdb. Studies of this project are undoubtedly of notable interest to many biologists given the complex genetic, biochemical, physiological and evolutionary processes that remain at the heart of host-pathogen interactions. In addition, we will continue to collect known *C. sinensis* full-length cDNAs to update the database for public use. The annotated functional protein presented herein and our knowledge database will be useful for parasite researchers who wish to clone and confirm full-length *C. sinensis* cDNAs of interest.

## Availability

The ClonorESTdb is open and freely available. All questions, comments and requests should be sent via email to todaewon@gmail.com.

**Project name:** ClonorESTdb.

**Project home page:**http://pathod.cdc.go.kr/clonorestdb/.

**Operating system:** Linux.

**Programming languages:** HTML, JSP, CSS3, JavaScript, AJAX, Oracle.

**Other requirements:** none.

**License:** None required.

## Competing interests

The authors declare that they have no competing interests.

## Authors’ contributions

DK and WY designed and implemented the database and website and wrote the manuscript, and DK, WY and SL developed the web interfaces, assisted with the design of the database and performed database system administration. SC, YK and ML helped with the preparation of the EST data sets (sample collection, cDNA library construction and sequencing). WL and JJ served as the principal investigators of the project. All authors contributed to the writing of the manuscript and have read and approved the final submitted version.

## References

[B1] Lun ZR Gasser RB Lai DH Li AX Zhu XQ Yu XB Fang YY Clonorchiasis: a key foodborne zoonosis in China Lancet Infect Dis 2005 5 31 41 15620559 10.1016/S1473-3099(04)01252-6

[B2] Keiser J Utzinger J Emerging foodborne trematodiasis Emerg Infect Dis 2005 11 1507 1514 16318688 10.3201/eid1110.050614 PMC3366753

[B3] Kaewkes S Taxonomy and biology of liver flukes Acta Trop 2003 88 177 186 14611872 10.1016/j.actatropica.2003.05.001

[B4] Kim TI Na BK Hong SJ Functional genes and proteins of *Clonorchis sinensis* Korean J Parasitol 2009 47 Suppl S59 S68 19885336 10.3347/kjp.2009.47.S.S59 PMC2769219

[B5] Sithithaworn P Haswell-Elkins MR Mairiang P Satarug S Mairiang E Vatanasapt V Elkins DB Parasite-associated morbidity: liver fluke infection and bile duct cancer in northeast Thailand Int J Parasitol 1994 24 833 843 7982745 10.1016/0020-7519(94)90009-4

[B6] Marcos LA Terashima A Gotuzzo E Update on hepatobiliary flukes: fascioliasis, opisthorchiasis and clonorchiasis Curr Opin Infect Dis 2008 21 523 530 18725803 10.1097/QCO.0b013e32830f9818

[B7] Lim JH Radiologic findings of clonorchiasis AJR Am J Roentgenol 1990 155 1001 1008 2120925 10.2214/ajr.155.5.2120925

[B8] Bouvard V Baan R Straif K Grosse Y Secretan B El Ghissassi F Benbrahim-Tallaa L Guha N Freeman C Galichet L Cogliano V WHO International Agency for Research on Cancer Monograph Working Group A review of human carcinogens–Part B: biological agents Lancet Oncol 2009 10 321 322 19350698 10.1016/s1470-2045(09)70096-8

[B9] Margulies M Egholm M Altman WE Attiya S Bader JS Bemben LA Berka J Braverman MS Chen YJ Chen Z Dewell SB Du L Fierro JM Gomes XV Godwin BC He W Helgesen S Ho CH Irzyk GP Jando SC Alenquer ML Jarvie TP Jirage KB Kim JB Knight JR Lanza JR Leamon JH Lefkowitz SM Lei M Li J Genome sequencing in microfabricated high-density picolitre reactors Nature 2005 437 376 380 16056220 10.1038/nature03959 PMC1464427

[B10] Bentley DR Balasubramanian S Swerdlow HP Smith GP Milton J Brown CG Hall KP Evers DJ Barnes CL Bignell HR Boutell JM Bryant J Carter RJ Keira Cheetham R Cox AJ Ellis DJ Flatbush MR Gormley NA Humphray SJ Irving LJ Karbelashvili MS Kirk SM Li H Liu X Maisinger KS Murray LJ Obradovic B Ost T Parkinson ML Pratt MR Accurate whole human genome sequencing using reversible terminator chemistry Nature 2008 456 53 59 18987734 10.1038/nature07517 PMC2581791

[B11] Shendure J Porreca GJ Reppas NB Lin X McCutcheon JP Rosenbaum AM Wang MD Zhang K Mitra RD Church GM Accurate multiplex polony sequencing of an evolved bacterial genome Science 2005 309 1728 1732 16081699 10.1126/science.1117389

[B12] Berriman M Haas BJ LoVerde PT Wilson RA Dillon GP Cerqueira GC Mashiyama ST Al-Lazikani B Andrade LF Ashton PD Aslett MA Bartholomeu DC Blandin G Caffrey CR Coghlan A Coulson R Day TA Delcher A DeMarco R Djikeng A Eyre T Gamble JA Ghedin E Gu Y Hertz-Fowler C Hirai H Hirai Y Houston R Ivens A Johnston DA The genome of the blood fluke *Schistosoma mansoni* Nature 2009 460 352 358 19606141 10.1038/nature08160 PMC2756445

[B13] Zhou Y Zheng H Chen Y Zhang L Wang K Guo J Huang Z Zhang B Huang W Jin K Dou T Hasegawa M Wang L Zhang Y Zhou J Tao L Cao Z Li Y Vinar T Brejova B Brown D Li M Miller DJ Blair D Zhong Y Chen Z Liu F Hu W Wang ZQ Zhang QH The *Schistosoma japonicum* genome reveals features of host-parasite interplay Nature 2009 460 345 351 19606140 10.1038/nature08140 PMC3747554

[B14] Yoo WG Kim DW Ju JW Cho PY Kim TI Cho SH Choi SH Park HS Kim TS Hong SJ Developmental transcriptomic features of the carcinogenic liver fluke, *Clonorchis sinensis* PLoS Negl Trop Dis 2011 5 e1208 21738807 10.1371/journal.pntd.0001208 PMC3125140

[B15] Ewing B Hillier L Wendl MC Green P Base-calling of automated sequencer traces using phred. I. Accuracy assessment Genome Res 1998 8 175 185 9521921 10.1101/gr.8.3.175

[B16] Jurka J Kapitonov VV Pavlicek A Klonowski P Kohany O Walichiewicz J Repbase Update, a database of eukaryotic repetitive elements Cytogenet Genome Res 2005 110 462 467 16093699 10.1159/000084979

[B17] Pertea G Huang X Liang F Antonescu V Sultana R Karamycheva S Lee Y White J Cheung F Parvizi B Tsai J Quackenbush J TIGR Gene Indices clustering tools (TGICL): a software system for fast clustering of large EST datasets Bioinformatics 2003 19 651 652 12651724 10.1093/bioinformatics/btg034

[B18] Huang X Madan A CAP3: A DNA sequence assembly program Genome Res 1999 9 868 877 10508846 10.1101/gr.9.9.868 PMC310812

[B19] Benson G Tandem repeats finder: a program to analyze DNA sequences Nucleic Acids Res 1999 27 573 580 9862982 10.1093/nar/27.2.573 PMC148217

[B20] Barker G Batley J O' Sullivan H Edwards KJ Edwards D Redundancy based detection of sequence polymorphisms in expressed sequence tag data using autoSNP Bioinformatics 2003 19 421 422 12584131 10.1093/bioinformatics/btf881

